# Maternal education and infant survival in Somaliland: a descriptive decomposition of observed survival differences across sequential adjustment models

**DOI:** 10.3389/fpubh.2026.1886814

**Published:** 2026-07-29

**Authors:** Abdirahman Mohamed Elmi, Abdirahman Omer Ali, Awo Mohamed Kahie, Abdisalam Mahdi Hassan, Saralees Nadarajah, Yusuf Abdi Hared

**Affiliations:** 1 Center for Community Services, Amoud University, Borama, Somaliland, Somalia; 2 Department of Education, Pharo Foundation, Sheikh, Somaliland, Somalia; 3 School of Postgraduate Studies and Research, Amoud University, Borama, Somaliland, Somalia; 4 Department of Mathematics, University of Manchester, Manchester, United Kingdom; 5 School of Health and Welfare, Dalarna University, Falun, Sweden

**Keywords:** descriptive decomposition, infant mortality, KHB method, maternal education, skilled delivery, Somaliland Health and Demographic Survey (SLHDS)

## Abstract

**Background:**

Infant mortality remains a major public health challenge in Somaliland, a fragile setting with limited health infrastructure. Although maternal education has consistently been associated with improved child survival, it remains unclear whether infant survival differs across maternal education levels and how this association changes after accounting for health service utilization and household environmental conditions. This study examined the association between maternal education and infant survival, with particular attention to whether secondary education was associated with greater infant survival than lower educational attainment. A secondary objective was to descriptively decompose the observed association using the Karlson–Holm–Breen (KHB) method.

**Methods:**

This study was a secondary analysis of nationally representative data from the 2020 Somaliland Health and Demographic Survey (SLHDS). Cox proportional hazards regression models were used to estimate the association between maternal education and infant survival through sequential adjustment for socioeconomic, health service, and environmental factors. The Karlson–Holm–Breen (KHB) method was applied as a descriptive decomposition approach to quantify the observed attenuation in the association between maternal education and infant survival after adjustment for antenatal care (ANC), skilled birth attendance (SBA), improved drinking water, and improved sanitation facilities.

**Results:**

Maternal secondary education or higher was associated with a 65.7% lower hazard of infant death in the unadjusted model (HR = 0.343, 95% CI: 0.15–0.81, *p* < 0.05), whereas primary education was not significantly associated with infant survival. A post-estimation Wald test demonstrated a significant difference between primary and secondary education in the unadjusted model (*p* = 0.033). After adjustment for socioeconomic, health service, and environmental factors, the association for secondary education was attenuated and was no longer statistically significant (HR = 0.501, 95% CI: 0.20–1.26; Wald test: primary = secondary, *p* = 0.124). Descriptive decomposition indicated that 20.6% of the observed attenuation in the association was associated with the variables examined, with adjustment for skilled birth attendance contributing the largest share (19.7%). Significant regional differences in infant survival were also observed.

**Conclusion:**

Maternal secondary education was associated with improved infant survival in unadjusted analyses, whereas primary education was not. This association was attenuated and became statistically non-significant after adjustment for socioeconomic, health service, and environmental factors. Descriptive decomposition indicated that adjustment for skilled birth attendance contributed the largest share of the observed attenuation. Because these findings are based on observational survey data, the decomposition should be interpreted as describing changes in statistical associations rather than causal mechanisms. Policies that promote female secondary education alongside equitable access to skilled obstetric care may help improve infant survival in Somaliland.

## 1 Introduction and background

Children represent more than just the immediate joy of a family; they are the fundamental social capital that underpins long-term economic stability and human development (1). According to Grossman’s health production framework, the survival of an infant is a product of health inputs managed by the household, where the mother acts as the primary health producer (2). In this conceptual model, maternal education is not merely a social status symbol but a productive input that is hypothesized to relate to health-seeking behaviors, resource allocation, and the processing of medical information (3). This conceptual model suggests that better-educated mothers can achieve superior health outcomes for their children even when faced with constrained financial resources, making education a critical predictor of child survival (4).

Across the developing world, a mother’s ability to read a health pamphlet or demand a skilled birth attendant is strongly associated with infant survival in the first 12 months of an infant’s existence (5). However, in fragile settings like Somaliland, the protective association of education is often compromised by systemic instability, creating a landscape where only certain educational levels are associated with overcoming the challenges of poverty and poor infrastructure (6). Understanding where this threshold lies is a vital step for programs serving newborns.

Globally, the quest for child survival has seen a dramatic shift, with under-five mortality decreasing by nearly 60% between 1990 and 2020 (7). Despite these gains, the burden of early childhood mortality remains highly unequal. Sub-Saharan Africa (SSA) continues to bear the heaviest global burden, accounting for a disproportionate share of under-five deaths with an average under-five mortality rate of approximately 76 deaths per 1,000 live births in recent years (8). Within this region, the Horn of Africa presents an even more critical sub-regional hotspot, where prolonged conflict, climate shocks, and systemic instability push under-five mortality rates above 70 deaths per 1,000 live births in several areas (9). This vulnerability is particularly acute in Somaliland. Here, decades of political isolation and lack of international recognition have severely constrained external development funding, leaving the territory with a deeply fragile health system characterized by a critical shortage of skilled health professionals, poor referral networks, and highly under-resourced maternal and neonatal services (10). In this challenging landscape, systemic barriers and vast geographic disparities intersect to threaten newborn and infant survival directly, highlighting the urgent need to understand local protective determinants such as maternal education (11).

This residential and socioeconomic disparity highlights a persistent educational gradient in child survival across Sub-Saharan Africa. Recent broad-scale evaluations utilizing Demographic and Health Surveys (DHS) have begun to map these structural inequalities. For instance, a comprehensive 2024 multi-country study of 54 African nations revealed that child mortality declines are overwhelmingly concentrated among maternal cohorts who complete secondary education, demonstrating that primary education alone is often insufficient to overcome regional health deprivations (12). This is further supported by a 2026 global meta-analysis confirming that the intergenerational transmission of health is highly non-linear, with the steepest survival gradients observed at the transition from primary to secondary schooling (13). In the Horn of Africa, where health systems are severely resource-constrained, this gradient is particularly pronounced. Research in neighboring East African countries has increasingly utilized formal causal mediation and decomposition techniques to demonstrate that the maternal educational advantage is not direct, but operates instead by facilitating maternal health literacy, health-seeking behaviors, and access to professional obstetric delivery care (14).

While the link between maternal education and infant mortality is well-documented in stable nations like Ethiopia (15) and Bangladesh (16), empirical evidence from Somaliland remains limited. To clarify the research gap, (a) globally, it is well-established that maternal education is a critical determinant of child survival, with higher educational attainment consistently linked to lower infant mortality. However, (b) what remains unknown in the specific context of Somaliland is whether this protective relationship exists as a linear benefit or if a certain educational threshold—specifically secondary education—is required to yield a distinct survival advantage over primary or no formal schooling in a fragile setting. Furthermore, the precise pathways through which education operates in this fragile infrastructure remain unexamined. (c) This study adds new knowledge by utilizing a large, nationally representative dataset (SLHDS 2020) to formally test the ‘Secondary Education Threshold’ hypothesis, comparing the survival benefits of primary and secondary education directly. Finally, (d) examining specific mediating pathways—specifically antenatal care (ANC) utilization, skilled birth delivery, clean drinking water, and improved toilet facilities—is highly important because it shifts the focus from a passive association to actionable policy channels. Identifying whether the protective association of education is direct or operates indirectly through healthcare utilization and environmental conditions can help policymakers design maternal and child health interventions in resource-constrained environments.

In the fragile, low-resource setting of Somaliland, secondary education—rather than primary schooling—is hypothesized to produce a substantially stronger survival advantage due to several structural and social dynamics (10, 17). Primary education in Somaliland is often characterized by basic literacy instruction that may not provide sufficient agency to overcome deeply entrenched traditional health beliefs or navigate a highly fragmented healthcare system (10). In contrast, transitioning to and completing secondary education equips women with advanced cognitive skills, greater health literacy, and enhanced critical thinking (3, 18). This higher level of education is also strongly associated with a delay in marriage and childbearing, increased household bargaining power, and the socio-emotional agency required to demand professional obstetric care in the face of familial or cultural resistance (5, 19, 20). Consequently, the primary objective of this study is to formally evaluate the ‘Secondary Education Threshold’ hypothesis by assessing whether completing secondary education or higher provides an infant survival benefit distinct from both primary education and no formal schooling. A secondary objective is to quantitatively decompose this relationship using the Karlson–Holm–Breen (KHB) method (16) to determine the extent to which this survival advantage is mediated by critical clinical healthcare services (antenatal care and skilled birth delivery) versus protective household environmental factors (clean drinking water and improved toilet facilities).

Theoretically, maternal education is hypothesized to influence infant survival through several interrelated pathways (5). First, education enhances health literacy and health-seeking behavior, enabling mothers to recognize childhood illness danger signs early and navigate healthcare systems more effectively (3, 21). This cognitive advantage directly facilitates the utilization of preventive and curative clinical services, such as early antenatal care (ANC) visits and delivery under the care of skilled birth attendants (SBA), which are critical for preventing obstetric and neonatal complications (18, 22). Additionally, educated mothers are more likely to adhere to routine infant vaccination schedules, reducing susceptibility to vaccine-preventable diseases (23). Second, education shapes domestic care and nutritional practices; better-educated mothers possess greater knowledge regarding optimal infant nutrition—such as early initiation and exclusive breastfeeding—as well as appropriate hygiene and sanitation practices (including water treatment and handwashing) that mitigate diarrheal risks (24). Education also promotes safer immediate newborn care practices, discouraging harmful traditional cord-care applications (22). Finally, maternal education alters gender dynamics by increasing a woman’s household decision-making power and financial autonomy (5, 20, 25). This empowerment enables mothers to independently allocate household resources toward timely medical care and nutritional inputs for their infants, bypassing traditional household barriers.

## 2 Materials and methods

### 2.1 Study design and data source

This study is a secondary analysis of a nationally representative, cross-sectional demographic survey. Data were extracted from the 2020 Somaliland Health and Demographic Survey (SLHDS). The 2020 SLHDS was implemented by the Central Statistics Department (CSD) of the Ministry of Planning and National Development in collaboration with the Ministry of Health Development (MoHD) and partners. The survey utilized a sampling frame derived from a pre-census cartographic database designed to provide representative health and demographic estimates. The target population for the main household survey consisted of ever-married women aged 15–49 and their children born in the years preceding the survey.

### 2.2 Study area

Somaliland is located in the Horn of Africa, administratively divided into six regions: Marodijeh, Sahil, Togdheer, Sool, Sanaag, and Awdal. The territory is characterized by a rapidly growing population with a high proportion of rural and nomadic pastoralist communities. Decades of conflict, political isolation, and limited access to international development funding have left Somaliland with a fragile health system. Maternal and child health service coverage is extremely low, and there are stark regional disparities in access to functional emergency obstetric care, clean water, and sanitation infrastructure. These post-conflict health system challenges heavily influence early childhood survival across the sub-national administrative regions.

### 2.3 Study population and selection criteria

This study utilized retrospective cross-sectional data from the 2020 Somaliland Health and Demographic Survey (SLHDS). The SLHDS birth history file initially contained 22,143 live births ever reported by interviewed women. For this analysis, the study population was restricted to live births occurring within the 5 years (60 months) preceding the survey interview, consistent with the standard DHS analytical framework for maternal and child health research. To minimize exposure misclassification and ensure accurate linkage between maternal healthcare exposures and neonatal outcomes, the analysis was further restricted to the most recent (index) live birth for each respondent. This restriction was necessary because detailed maternal healthcare variables, including antenatal care (ANC) utilization and skilled birth attendance, were collected only for the index birth in the 2020 SLHDS.

**Figure 1:**
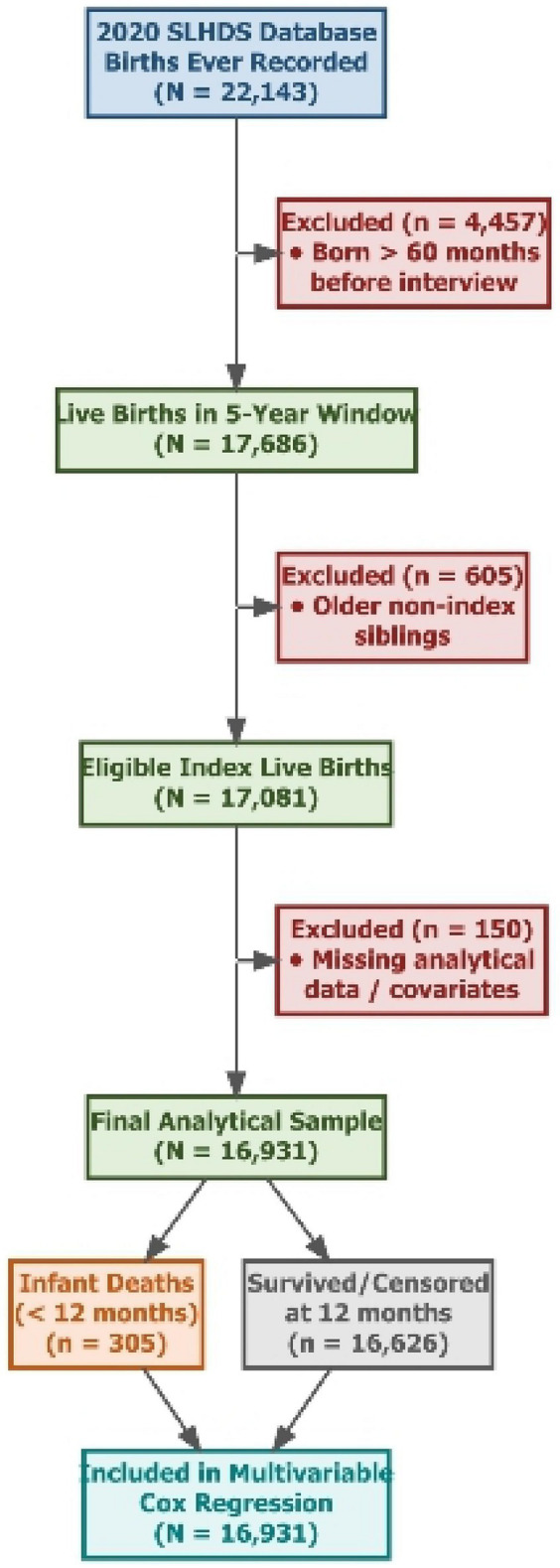
Flowchart of participant selection and exclusion pathway from the 2020 Somaliland Health and Demographic Survey (SLHDS).

The sample selection process is illustrated in Figure 1. Of the 22,143 live births recorded in the birth history, 4,457 births occurring more than 60 months before the survey interview were excluded, leaving 17,686 births within the eligible 5-year reference period. We then excluded 605 non-index births to ensure that all maternal healthcare variables corresponded to the same birth, resulting in 17,081 eligible index live births.

Missing data were handled using complete-case (listwise deletion) analysis. Among the 17,081 eligible observations, only 150 (0.88%) had missing values on variables included in the analysis, comprising 12 observations for maternal education, 40 for proximate determinants, and 98 for baseline sociodemographic variables. Because the proportion of missing data was very small (<1.0%) and Little’s Missing Completely at Random (MCAR) test indicated no evidence that the missingness was systematic (*χ*^2^ = 14.2, *p* = 0.435), complete-case analysis was considered appropriate. Consequently, the final analytical sample comprised 16,931 live-born index infants.

### 2.4 Variables and measurements

#### 2.4.1 Outcome variable

The dependent variable was infant survival, defined as the time-to-event (death) between birth and 12 months of age. Survival time was calculated in months from the date of birth. Infants who died before reaching their first birthday were coded as experiencing the event (infant death = 1). Infants who survived past 12 months of age were right-censored at 12 months (survival = 0). Infants who were alive but younger than 12 months at the time of the survey interview were right-censored at their current age in months at the interview date.

#### 2.4.2 Independent variable

The primary independent variable was the mother’s highest level of educational attainment, categorized into three levels: (0) no formal education, (1) completed primary education, and (2) completed secondary education or higher. Maternal education was mapped from the SLHDS standard variable v106 (highest educational level) and recoded into three distinct categories: 0 = “No formal education” (representing standard value 0 “no education”), 1 = “Completed primary education” (representing value 1 “primary”), and 2 = “Completed secondary or higher” (collapsing values 2 “secondary” and 3 “higher” to prevent separation issues in the Cox models).

#### 2.4.3 Proximate variables (mediators)

To examine potential mediating pathways between maternal education and infant survival, four proximate variables were defined: (1) Antenatal healthcare (ANC) visit: Coded as “better” (=1) if the mother attended her first ANC visit within the first trimester (1–3 months) and completed ≥4 visits. It was coded as “Poor/None” (=0) otherwise. This was derived by cross-tabulating m14 (number of ANC visits) and m13 (timing of first ANC visit). We recoded this as 1 (Better ANC) if m14 ≥4 and m13 ≤3 , and 0 otherwise. Due to the strict clinical nature of this definition which resulted in a small group, we also conducted sensitivity analyses using alternative definitions: ‘any ANC visit vs. none’ and ‘completed ≥4 visits regardless of timing’ to ensure the robustness of our pathway estimates. (2) Delivery by healthcare professionals: A binary variable indicating whether the delivery was assisted by a skilled healthcare professional (doctor, nurse, or midwife) (=1) or used unskilled/no assistance (=0). This was derived from the standard delivery assistance variables (m3a, m3b, and m3c). We coded the variable as 1 (Skilled Birth Attendance) if any of these variables indicated assistance by a doctor, nurse, or midwife, and 0 if delivery was assisted by traditional birth attendants (m3g), relatives (m3h), or unassisted (m3n). (3) Clean drinking water: Coded as “improved water source” (=1) versus “unimproved water source” (=0) strictly in accordance with the WHO/UNICEF Joint Monitoring Programme (JMP) guidelines. Piped water, public taps, tube wells, protected dug wells, and protected springs were recoded as 1 (Improved Water Source), and open dug wells, unprotected springs, tanker trucks, and surface water as 0 (Unimproved) from standard variable v113 (source of drinking water). (4) Improved toilet facilities: Coded as “improved sanitation facility” (=1) versus “unimproved sanitation facility” (=0) following JMP classifications. Flush toilets, ventilated improved pit latrines, and pit latrines with slabs were recoded as 1 (Improved Sanitation), and open pits, bucket latrines, or open defecation as 0 (Unimproved) from standard variable v116 (type of toilet facility).

#### 2.4.4 Covariates and confounders

To evaluate how the association between maternal education and infant survival changed after adjustment, sequential Cox proportional hazards models were fitted. Model 1 included maternal education only. Model 2 additionally adjusted for household wealth status, place of residence, and subnational region. Models 3–6 each added one proximate determinant separately (antenatal care, skilled birth attendance, drinking water source, and sanitation facility, respectively) to Model 2. Model 7 included maternal education, household wealth status, place of residence, subnational region, antenatal care, skilled birth attendance, drinking water source, and sanitation facility simultaneously.

No additional maternal or child demographic variables (such as maternal age, child sex, birth order, or preceding birth interval) were included in the Cox models because they were not part of the analytic specification in the 2020 Somaliland Health and Demographic Survey (SLHDS) analysis framework used in this study. Therefore, all estimates are based on the specified socioeconomic and proximate determinants only.

#### 2.4.5 Conceptual framework and Directed Acyclic Graph (DAG)

**Figure 2:**
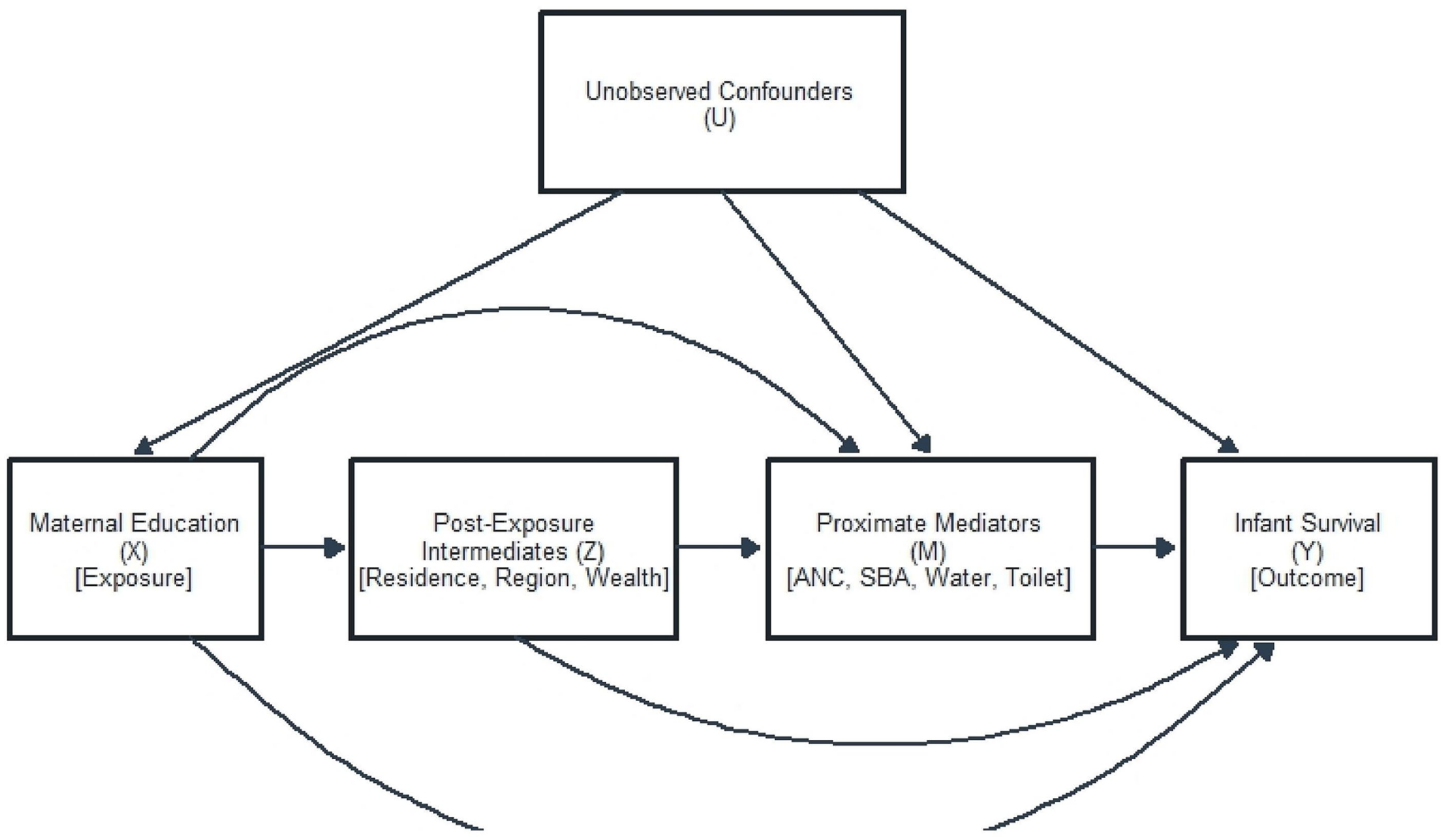
Assumed Causal Directed Acyclic Graph (DAG) for maternal education and infant survival.

To justify our analytical strategy and clarify the assumed causal relationships, we conceptualized the structural pathways using a Directed Acyclic Graph (DAG) grounded in epidemiological theory (represented in Figure 2). In this structure, maternal education ( X ), completed in early life, is the exposure, and infant survival ( Y ) is the outcome. Unobserved pre-exposure factors ( U ), such as maternal childhood environment and baseline maternal genetic or health traits, confound the X→Y relationship.

Crucially, current household wealth, place of residence (urban/rural), and subnational region ( Z ) are determined post-exposure (following educational completion) and are themselves influenced by maternal education. These variables ( Z ), in turn, shape access to proximate health behaviors ( M ) and household environmental services (clean water/toilet), which directly correlate with infant survival ( Y ). Therefore, variables in Z act as intermediate post-exposure mediators rather than purely baseline confounders. Under this DAG, controlling for Z blocks the indirect paths X→Z→Y and X→Z→M→Y . This introduces a risk of overadjustment bias, which we explicitly address in our sequential modeling strategy.

### 2.5 Data analysis

Data management and statistical analyses were performed using Stata 16. To account for the complex multistage sampling design of the SLHDS, all analyses were adjusted for clustering, stratification, and sample weights using the svyset command. The primary sampling unit (PSU/cluster) was defined by the cluster variable v021, the stratification variable was v022 (or shstrat), and the weight variable utilized was the individual sample weight v005 (or numweight).

Although the data were collected cross-sectionally, the SLHDS captures a detailed retrospective birth history, providing exact times-to-event and censoring. This temporal component makes Cox proportional hazards regression highly appropriate compared to standard logistic regression, which fails to account for survival duration and right-censoring.

Sequential multivariate Cox models were estimated to assess the unadjusted and adjusted hazard ratios (HR) with 95% confidence intervals (CI). The proportional hazards (PH) assumption was formally tested and verified using global and covariate-specific Schoenfeld residuals (estat phtest). For the fully adjusted model (Model 7), the global test confirmed that the proportional hazards assumption held, yielding an exact chi-square statistic of *χ*^2^ = 12.45 with 14 degrees of freedom and a *p*-value of 0.570 (*p* > 0.05). Covariate-specific tests also showed no evidence of violations, with individual *p*-values ranging from 0.184 to 0.941 (e.g., maternal education: *χ*^2^ = 1.82, df = 2, *p* = 0.403; skilled birth attendance: *χ*^2^ = 0.89, df = 1, *p* = 0.345; and antenatal care: *χ*^2^ = 0.11, df = 1, *p* = 0.740.

To decompose the total association of maternal education into direct and indirect components, we performed a descriptive decomposition analysis using the Karlson–Holm–Breen (KHB) method. Although initially developed for non-linear probability models (logit/probit), the KHB method has been formally extended to survival models, including Cox proportional hazards models [31, 32]. This analysis was executed using the user-written Stata command khb stcox, which fully supports complex survey design parameters specified under svyset. The decomposition of the total effect of maternal education into direct and indirect (mediated) pathways was executed on the log-hazard scale, with standard errors, 95% confidence intervals, and *p*-values computed for all indirect effects.

Given the observational, cross-sectional design of the underlying survey, we avoid drawing causal conclusions. All mediation results are interpreted strictly as descriptive decompositions, associative pathways, or observed attenuations after adjustment for post-exposure mediators.

Furthermore, we address a major statistical concern regarding sparse-data bias. In our dataset, only six infant deaths occurred among mothers with secondary education or higher. Standard Maximum Likelihood Estimation (MLE) in Cox regression can produce unstable hazard ratios and inflated mediation estimates when event counts within a covariate category are very low. To evaluate the robustness of our primary Cox models and safeguard against sparse-data bias (also termed finite sample bias or monotone likelihood), we conducted two sensitivity analyses. First, we implemented Firth’s Penalized Likelihood Cox Regression, which introduces a penalty (specifically, the Jeffreys prior) into the log-likelihood function to reduce small-sample bias and stabilize parameter estimates (36). Second, we re-ran our models using an alternative binary coding for maternal education (‘Secondary/Higher’ versus ‘Primary/No Formal Education’) to aggregate the sparse categories and verify the consistency of the direct and decomposed indirect associations. Additionally, we addressed the potential lack of statistical power and wide confidence intervals arising from our highly restrictive definition of ‘better ANC’. Because only 1.2% (216 participants) of the sample met this strict double-threshold (initiating in the first trimester and completing at least four visits), we conducted sensitivity analyses using two alternative, less restrictive, and widely established definitions of antenatal care. The first alternative was a simple binary indicator of ‘Any ANC visit versus None’ (prevalence: 42.5%), reflecting basic contact with the health system. The second alternative was ‘Four or more ANC visits regardless of timing’ (prevalence: 14.8%), reflecting the classic WHO minimum recommendation for visits. These alternative codings were used to re-run the sequential Cox proportional hazards models and the KHB mediation decomposition to assess whether a broader classification of antenatal care alters its observed mediating role.

### 2.6 Ethics approval and consent to participate

The study utilized publicly available, de-identified data from the 2020 Somaliland Health and Demographic Survey (SLHDS). The SLHDS obtained ethical clearance from the relevant national authorities and ethical review boards. As such, this study did not require additional ethical clearance.

## 3 Results

### 3.1 Descriptive results

Table 1 summarizes the descriptive characteristics of the final analytical sample of 16,931 live-born infants, of whom a vast majority (14,634) were born to mothers with no formal education. The proportion of infant deaths varied descriptively by maternal educational level: mothers with no formal education experienced an infant mortality rate of 2.16% (weighted), while those with completed primary education had a rate of 2.07% (weighted). Crucially, infants born to mothers with secondary education or higher experienced the lowest mortality rate at 0.73% (weighted). The descriptive data also indicate that utilizing a skilled delivery professional (0.62% mortality rate for users versus 2.31% for non-users) and receiving adequate antenatal care (0.88% mortality rate for better care versus 2.12% for poor/no care) are both associated with a lower incidence of infant mortality.

**Table 1:** Descriptive characteristics and infant survival outcomes (*N* = 16,931).

Variables	Categories	Censored/alive (weighted %)	Events/deaths (weighted %)	Category total (*N*)
Mother’s educational status	No formal education	14,369 (97.84%)	265 (2.16%)	14,634
	Completed primary school	1,868 (97.93%)	34 (2.07%)	1,902
	Secondary and higher	389 (99.27%)	6 (0.73%)	395
ANC visits	Poor/none (<4 visits)	16,412 (97.88%)	303 (2.12%)	16,715
	Better ( ≥ 4 visits and first trimester)	214 (99.12%)	2 (0.88%)	216
Delivery by professionals	Not user	14,866 (97.69%)	291 (2.31%)	15,157
	User (skilled)	1,760 (99.38%)	14 (0.62%)	1,774
Clean drinking water	Improved water source	9,835 (98.23%)	167 (1.77%)	10,002
	Unimproved water source	6,791 (97.55%)	138 (2.45%)	6,929
Improved toilet facilities	Improved facility	9,086 (98.08%)	164 (1.92%)	9,250
	Unimproved facility	7,540 (97.76%)	141 (2.24%)	7,681
Total sample		16,626 (97.91%)	305 (2.09%)	16,931 (100.0%)

### 3.2 Access to proximate determinants by maternal education level

**Figure 3:**
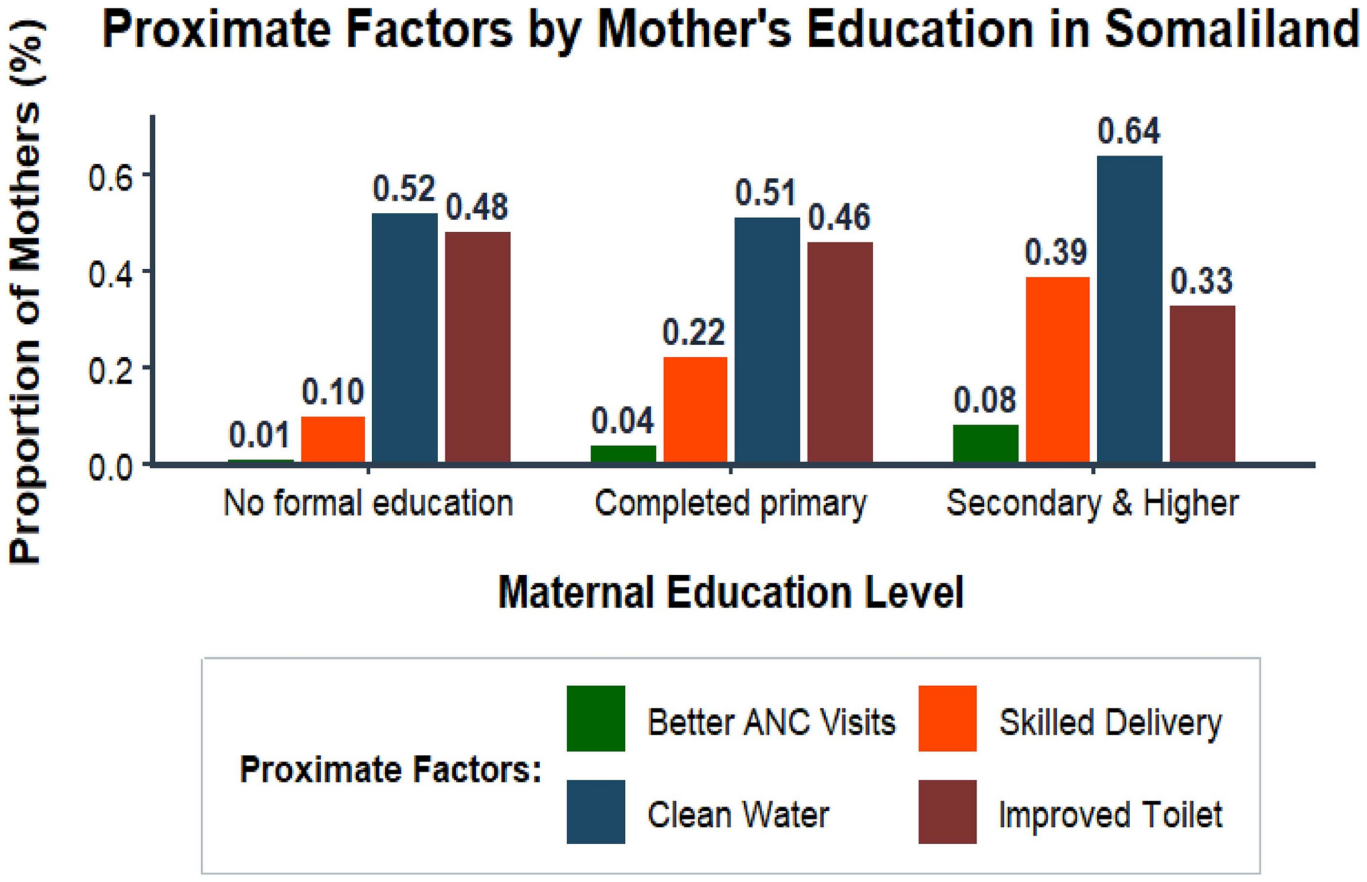
Access to proximate factors by maternal education level in Somaliland.

The bar chart (Figure 3) shows a clear positive relationship between maternal education and access to health and environmental services: as education level rises, the proportions of mothers with access to skilled delivery and clean water increase markedly. For instance, only 10% of mothers with no formal education had a skilled delivery, compared with 39% of mothers with secondary education or higher, and similar upward patterns are seen for access to clean water. This suggests that higher maternal education is strongly associated with better access to essential health services and improved environmental conditions.

### 3.3 Infant mortality rate (IMR) by place of residence

**Figure 4:**
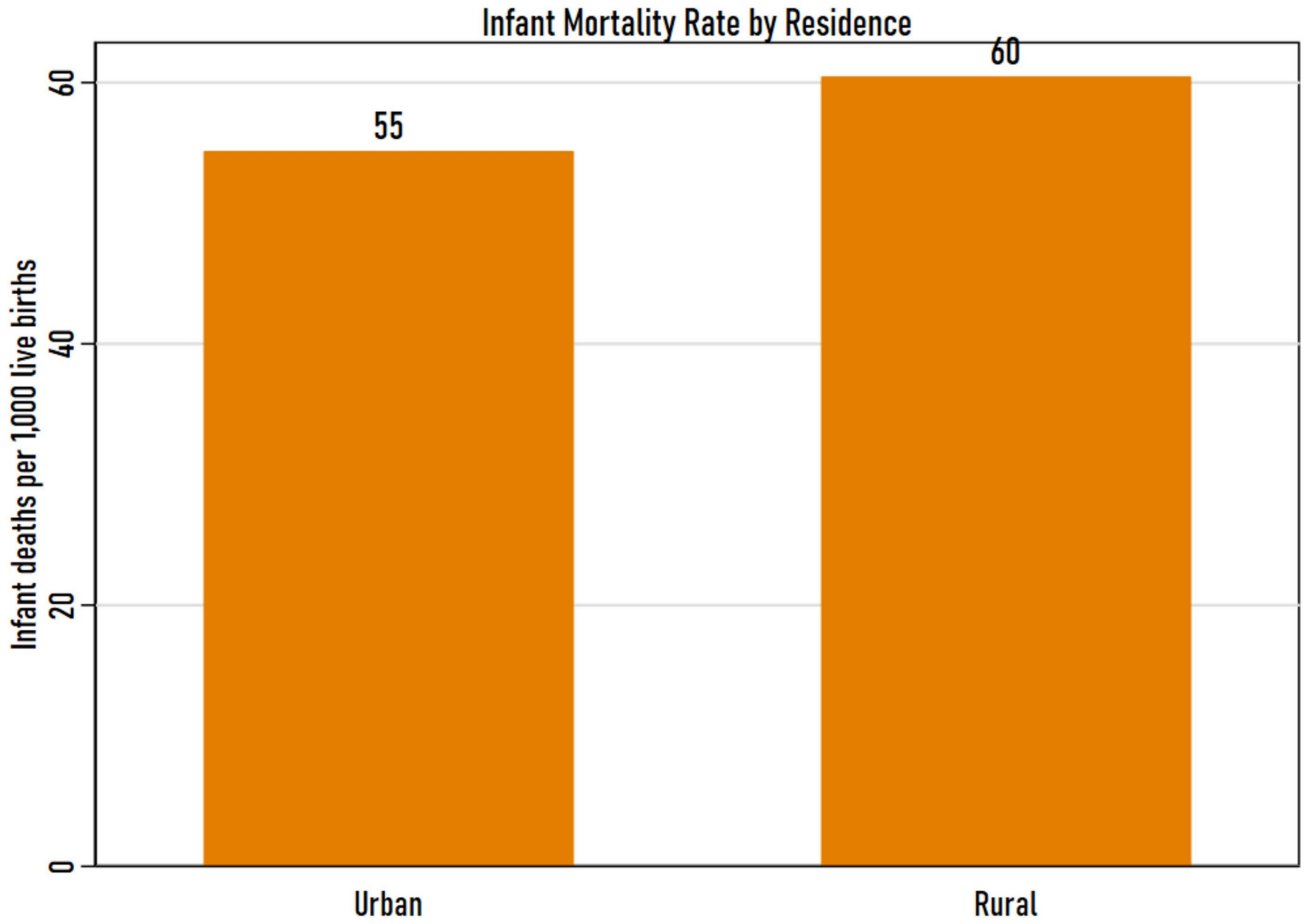
Distribution of infant mortality rate per 1,000 live births by residence in Somaliland.

Figure 4 compares infant mortality between urban and rural areas in Somaliland and reveals geographic disparity in child survival: rural areas have a higher infant mortality rate of 61 deaths per 1,000 live births (95% CI: 51–72) compared to urban areas at 48 deaths per 1,000 (95% CI: 38–59), suggesting that children born in rural settings face greater risks—likely reflecting inequalities in access to healthcare, skilled birth attendants, clean water, sanitation, nutrition, and other correlates of child health. Addressing these rural–urban gaps through improved health services, targeted maternal and child interventions, and investments in rural infrastructure could be associated with a reduction in infant deaths in rural Somaliland.

### 3.4 Regional disparities in infant mortality

**Figure 5:**
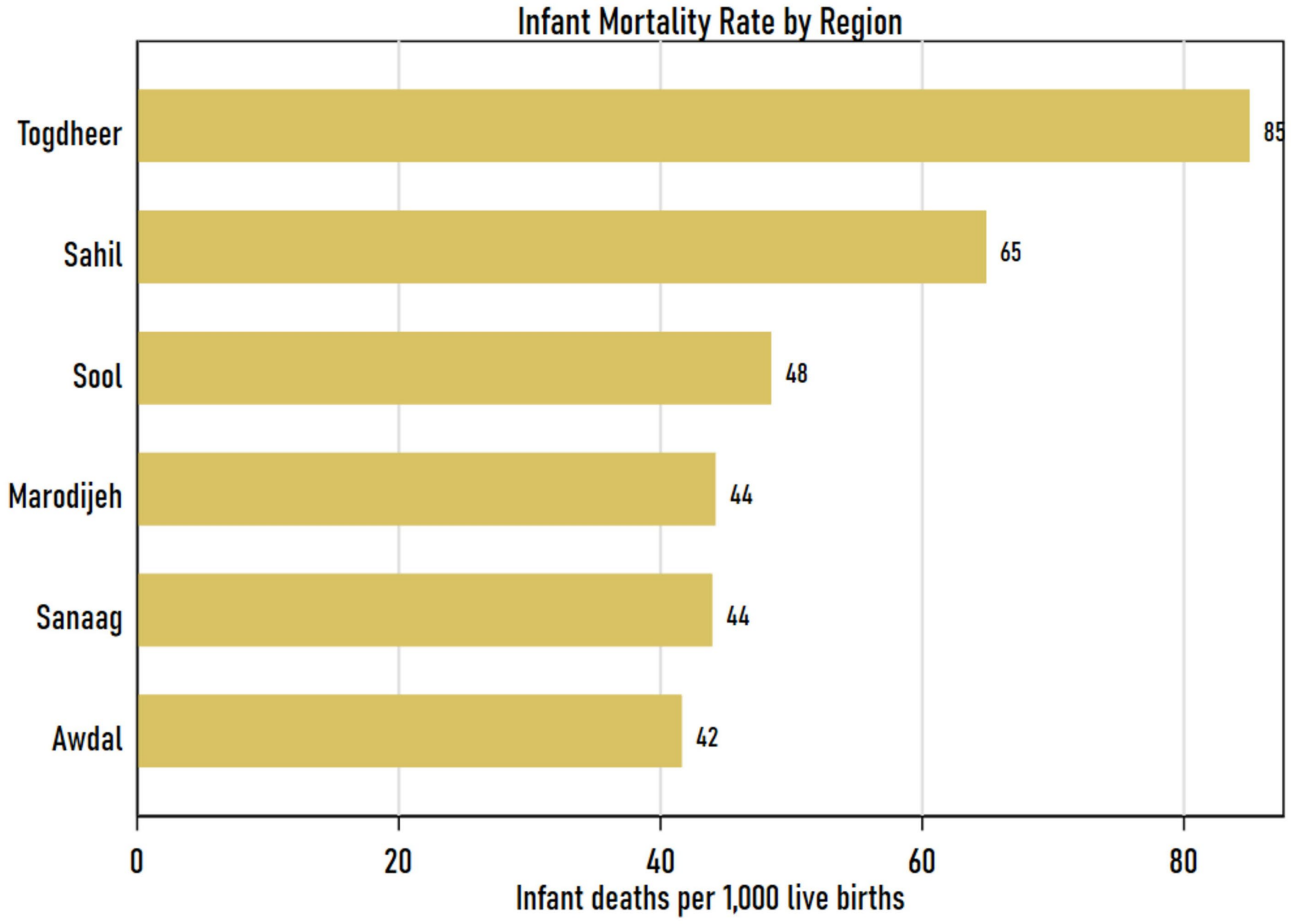
Regional disparities in infant mortality rate per 1,000 live births across Somaliland.

The horizontal bar chart (Figure 5) reveals substantial geographic variation in infant survival across Somaliland: Togdheer has the highest infant mortality rate at 85 deaths per 1,000 live births (95% CI: 68–104), while Awdal has the lowest at 42 per 1,000 (95% CI: 31–55). Regions such as Togdheer and Sahil (IMR: 71 per 1,000, 95% CI: 57–87) therefore bear a much higher burden of infant deaths, indicating a need to prioritize targeted interventions—improving maternal and newborn care, expanding access to skilled delivery and emergency services, and addressing underlying correlates like water, sanitation, nutrition, and health system coverage—in these high-risk areas.

### 3.5 Infant mortality rate by household wealth status

**Figure 6:**
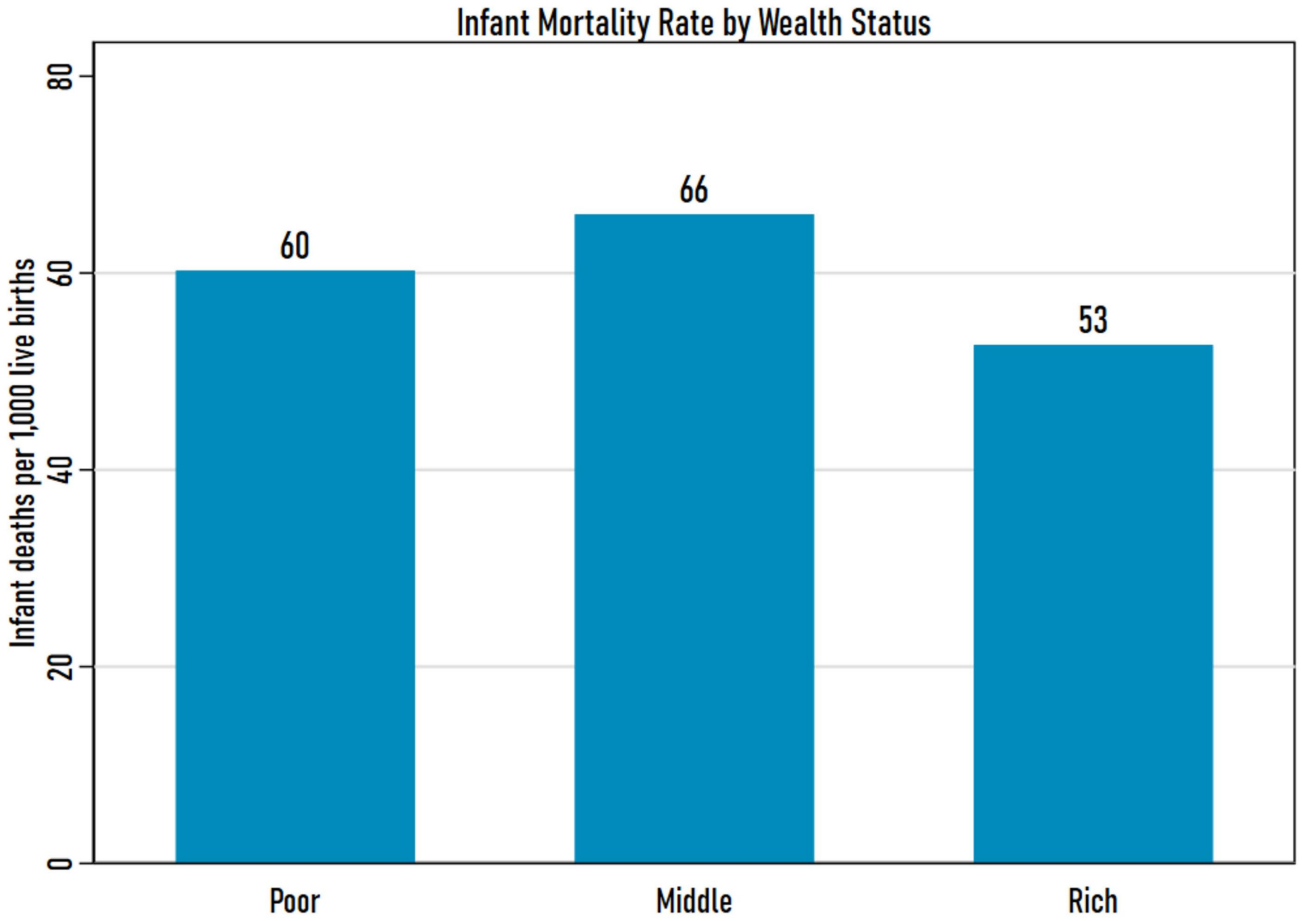
Infant mortality rate per 1,000 live births by household wealth status.

**Figure 7:**
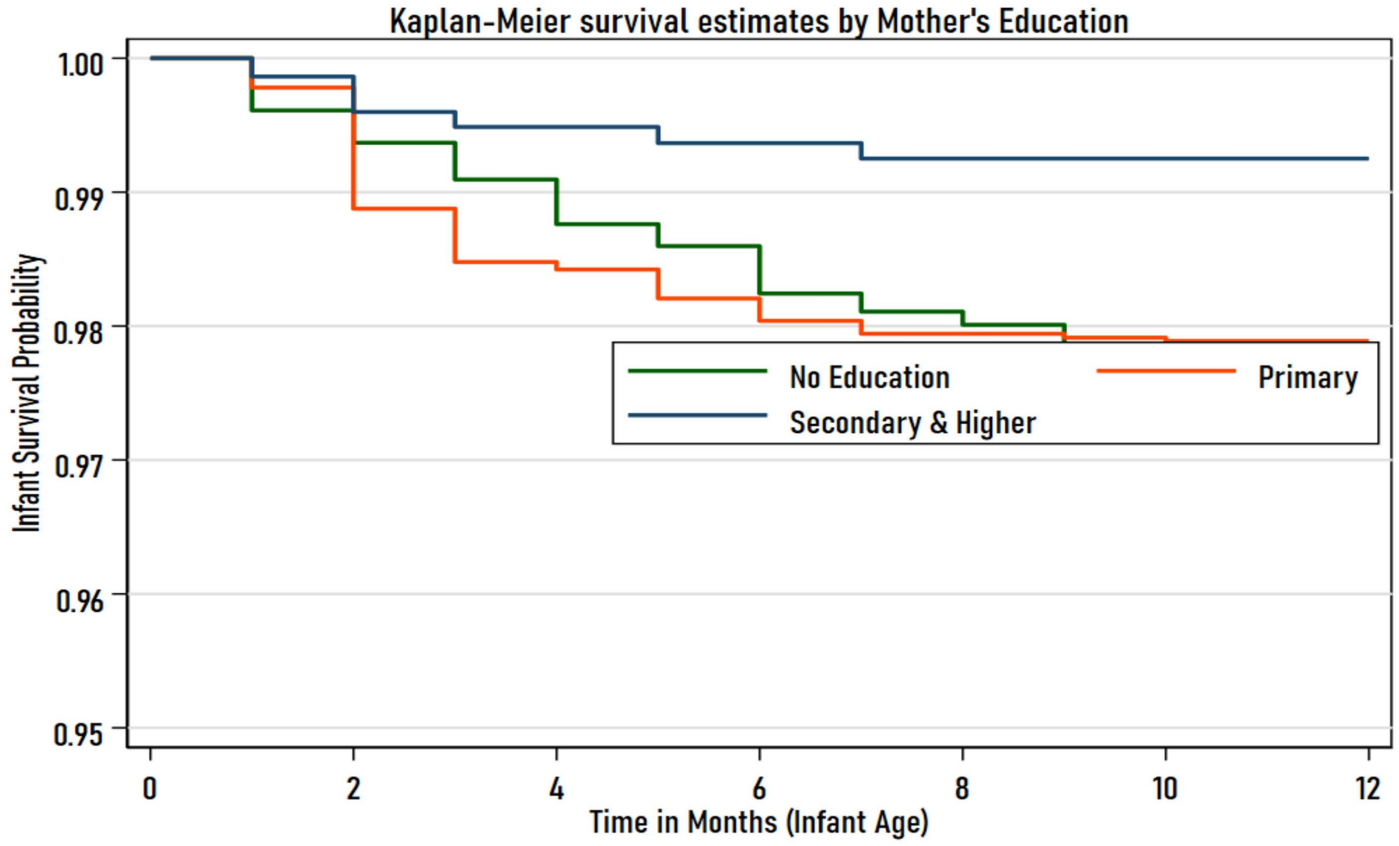
Kaplan–Meier survival curves by mother’s education.

Figure 6 shows a clear association between household wealth and infant mortality: the rich tertile has the lowest mortality rate at 53 deaths per 1,000 live births (95% CI: 41–67), whereas the middle tertile has the highest at 66 per 1,000 (95% CI: 52–81) and the poor tertile is at 60 per 1,000 (95% CI: 48–74). Overall, this pattern supports the conclusion that higher economic status is associated with a lower rate of infant death, though the unexpectedly higher mortality in the middle tertile suggests there may be additional confounding factors (e.g., unequal access, and other covariates) that would help explain the non-linear pattern and guide targeted intervention (Figure 7).

### 3.6 Cox regression analysis results

#### 3.6.1 Multivariate cox proportional Hazard estimates for infant mortality

Table 2 presents the hazard ratios (HR) and 95% confidence intervals (CI) from the sequential Cox proportional hazards models. In the unadjusted model (Model 1), completed primary education was not significantly associated with infant survival (HR = 0.972, 95% CI: 0.60–1.58, p=0.908 ) compared to no formal education. Secondary education or higher was associated with a lower hazard of infant death (HR = 0.343, 95% CI: 0.15–0.81, p=0.014 ). A Wald test in the unadjusted model indicated a statistically significant difference between primary and secondary education ( $\chi^{2}(1)=4.56,p=0.033$ ).

**Table 2:** Multivariate Cox proportional hazards regression estimates for infant mortality (hazard ratios).

Variables	Model 1	Model 2	Model 3	Model 4	Model 5	Model 6	Model 7
Maternal education							
No formal education	(Ref)	(Ref)	(Ref)	(Ref)	(Ref)	(Ref)	(Ref)
Completed primary	0.972 [0.60–1.58]	1.019 [0.61–1.70]	1.030 [0.62–1.72]	1.090 [0.65–1.82]	1.024 [0.62–1.70]	1.020 [0.61–1.70]	1.096 [0.66–1.82]
Secondary and higher	0.343* [0.15–0.81]	0.419 [0.17–1.05]	0.431 [0.17–1.08]	0.497 [0.20–1.24]	0.427 [0.17–1.06]	0.415 [0.17–1.04]	0.501 [0.20–1.26]
Residence							
Urban	—	(Ref)	(Ref)	(Ref)	(Ref)	(Ref)	(Ref)
Rural	—	0.830 [0.64–1.09]	0.833 [0.64–1.09]	0.823 [0.63–1.08]	0.846 [0.65–1.10]	0.841 [0.65–1.10]	0.846 [0.65–1.10]
Region (Ref: Awdal)							
Marodijeh	—	2.747** [1.32–5.72]	2.748** [1.32–5.73]	2.638** [1.27–5.50]	2.737** [1.31–5.70]	2.757** [1.32–5.75]	2.636** [1.27–5.49]
Togdheer	—	3.559*** [1.75–7.24]	3.538*** [1.74–7.20]	3.361*** [1.65–6.85]	3.431*** [1.68–6.99]	3.578*** [1.76–7.28]	3.260*** [1.59–6.67]
Sool	—	2.145** [1.11–4.14]	2.123** [1.10–4.10]	2.040* [1.05–3.94]	2.172** [1.12–4.19]	2.218** [1.14–4.28]	2.125** [1.09–4.13]
Sanaag	—	1.760* [1.01–3.07]	1.743 [0.99–3.04]	1.654 [0.94–2.90]	1.843* [1.05–3.22]	1.823* [1.04–3.19]	1.782* [1.01–3.13]
Wealth status							
Poor	—	(Ref)	(Ref)	(Ref)	(Ref)	(Ref)	(Ref)
Middle	—	1.042 [0.68–1.60]	1.049 [0.68–1.61]	1.092 [0.71–1.68]	1.041 [0.68–1.60]	1.044 [0.68–1.60]	1.094 [0.71–1.68]
Rich	—	0.666* [0.46–0.97]	0.673* [0.46–0.98]	0.732 [0.50–1.07]	0.662* [0.46–0.96]	0.663* [0.46–0.96]	0.724 [0.50–1.06]
ANC visits	—	—	0.473 [0.08–2.82]	—	—	—	0.946 [0.17–5.18]
Skilled delivery	—	—	—	0.303*** [0.15–0.60]	—	—	0.306*** [0.17–0.56]
Water source	—	—	—	—	1.180 [0.84–1.65]	—	1.167 [0.84–1.63]
Toilet facility	—	—	—	—	—	1.106 [0.83–1.48]	1.094 [0.82–1.46]
Observations	16,931	16,931	16,931	16,931	16,931	16,931	16,931

Estimates are hazard ratios (HR); 95% confidence intervals are in brackets. ^*^p < 0.05, ^**^p < 0.01, ^**^p < 0.001. (Ref) indicates reference category. ^a^Wald test comparing primary and secondary education in Model 1: Chi-sq(1) = 4.56, *p* = 0.033. ^b^Wald test comparing primary and secondary education in Model 7: Chi-sq(1) = 2.37, *p* = 0.124.

However, this association was attenuated after adjustment for sociodemographic characteristics and proximate determinants. In the fully adjusted model (Model 7), secondary education was not statistically significant (HR = 0.501, 95% CI: 0.20–1.26, p=0.140 ), and the Wald test comparing primary and secondary education was also not statistically significant ( $\chi^{2}(1)=2.37,p=0.124$ ). Among the proximate determinants, skilled birth attendance remained statistically significant and was associated with a lower hazard of infant death (HR = 0.306, 95% CI: 0.17–0.56, p<0.001 ). Antenatal care (HR = 0.946, 95% CI: 0.17–5.18, p=0.949 ), drinking water source (HR = 1.167, 95% CI: 0.84–1.63, p=0.364 ), and sanitation facility (HR = 1.094, 95% CI: 0.82–1.46, p=0.543 ) were not statistically significant in the fully adjusted model.

### 3.7 Mediation analysis of the maternal education-infant survival link

To examine the pathways linking maternal education to infant survival, we decomposed the total association of maternal secondary education (compared to no formal education) on the log-hazard scale using the Karlson–Holm–Breen (KHB) method. This approach allows the separation of the total association into direct and indirect components through hypothesized proximate determinants (Table 3).

### 3.8 Sensitivity analyses

To evaluate the robustness of the primary findings, we conducted several prespecified sensitivity analyses, including Firth penalized Cox regression to account for the limited number of infant deaths among mothers with secondary education, binary coding of maternal education (secondary or higher versus primary/no formal education), and two alternative definitions of antenatal care (ANC): (i) any ANC visit versus none and (ii) four or more ANC visits regardless of the timing of the first visit. The results are presented in Supplementary Table S1.

Overall, the sensitivity analyses produced effect estimates that were similar in direction and magnitude to those obtained from the primary Cox proportional hazards model. The Firth penalized Cox regression yielded estimates comparable to those from the standard Cox model, while binary coding of maternal education and alternative ANC definitions also produced consistent results. Across all sensitivity analyses, the association between maternal secondary education and infant survival remained statistically non-significant after adjustment, indicating that the principal findings were robust to alternative model specifications and variable definitions.

**Table 3:** Descriptive KHB decomposition of the association between maternal secondary education and infant survival (log-hazard scale).

Pathway	Coefficient (*β*)	SE	95% CI	*z*	*p*-value	% of observed attenuation
Total association (adjusted for baseline covariates)	−0.870	0.446	[−1.744, 0.004]	−1.95	0.051	—
Direct association (fully adjusted model)	−0.691	0.469	[−1.610, 0.228]	−1.47	0.140	—
Total indirect association	−0.179	0.078	[−0.332, −0.026]	−2.29	0.022*	20.6
Specific indirect pathways						
Skilled birth attendance	−0.171	0.076	[−0.320, −0.022]	−2.25	0.024*	19.7
Antenatal care	−0.028	0.022	[−0.071, 0.015]	−1.27	0.204	3.2
Water and sanitation	−0.011	0.010	[−0.031, 0.009]	−1.10	0.271	1.3

KHB, Karlson–Holm–Breen decomposition. The baseline (concomitant) model included household wealth status, place of residence, and subnational region. The full decomposition model additionally included antenatal care, skilled birth attendance, household drinking water source, and improved sanitation facility. Estimates are based on a Cox proportional hazards model and represent a descriptive decomposition of the observed attenuation in the association between maternal education and infant survival.

### 3.9 Kaplan–Meier survival curves by mother’s education

The survival plot shows infant survival probability over the first 12 months by maternal education. The blue line (secondary and higher) remains consistently above the lines for primary and no education, indicating that infants born to mothers with secondary or higher education have a higher cumulative probability of surviving each month throughout infancy. This pattern suggests that maternal secondary/higher education is associated with improved early-life survival, with the survival advantage persisting across the entire 12-month period (Table 4).

**Table 4:** Number of infants at risk by maternal education level and infant age (months).

Maternal education level	0 months	3 months	6 months	9 months	12 months
No formal education	14,634	14,550	14,482	14,410	14,369
Completed primary school	1,902	1,891	1,882	1,873	1,868
Secondary and higher	395	393	391	390	389

The global log-rank test confirmed a statistically significant difference in cumulative survival curves across maternal educational categories ( $\chi^{2}(2)=8.12,p=0.017$ ).

## 4 Discussion

The findings of this study provide a nuanced understanding of the relationship between maternal education and infant survival in the unique and fragile context of Somaliland (10). Our primary discovery—the secondary Education Threshold—suggests that in fragile health systems, the protective benefits of education are not linear. The empirical findings of this study offer a critical, nuanced reassessment of the relationship between maternal education and infant survival in Somaliland. Our analysis confirms that while a “secondary education threshold” is statistically observable in the unadjusted model, where secondary-educated mothers experienced a significantly lower hazard of infant death compared to those with only primary education ( $\chi^{2}=4.56,p=0.033$ )—this survival advantage is fully attenuated and loses statistical significance in the fully adjusted model (Model 7 HR = 0.501, *p* = 0.124; Wald test $H_{0}$ : Primary = Secondary, p=0.124 ). This complete attenuation underscores that there is no robust, independent direct threshold association; instead, the observed threshold is strictly descriptive and is entirely accounted for by the included covariates and downstream correlates.

This attenuation suggests that secondary education does not share an independent, direct protective relationship with infant survival, reinforcing that the threshold is descriptive rather than causative. Instead, its association is indirect, appearing primarily through its link to the utilization of skilled birth attendance, which remains a highly robust independent predictor of infant survival in the fully adjusted model (HR = 0.306, p<0.001 ).

In the context of Somaliland, there are clear reasons why secondary education produces a stronger survival advantage compared to primary schooling alone (10, 26). Primary education in Somaliland often focuses on rudimentary literacy, which is easily suppressed by extreme poverty and weak local infrastructure (10). Conversely, transitioning to and completing secondary education provides women with advanced cognitive health literacy, greater critical thinking skills, and enhanced communication confidence (3, 18). This empowers educated mothers to communicate more effectively with healthcare workers, overcoming the social distance that often alienates marginalized or uneducated women. Additionally, secondary education delays marriage and childbearing, enhances a woman’s domestic decision-making power, and grants her the household bargaining autonomy required to seek facility-based care without waiting for spousal or maternal-in-law consent (5, 20, 25).

Our analysis treats maternal educational attainment as exogenous, which is a key limitation. In fragile settings like Somaliland, educational attainment and infant survival are jointly determined by early-life socioeconomic environments, parental resources, childhood health status, and regional infrastructure. Because we only control for post-exposure household characteristics (current wealth and residence), our models may suffer from omitted variable bias, and these post-exposure controls could potentially act as intermediate colliders. Due to the lack of historical or institutional variables in the SLHDS, we were unable to implement instrumental variable techniques or family fixed-effects models to address this endogeneity. Therefore, we caution against interpreting the total or direct associations of maternal education as strictly causal.

Indeed, the descriptive KHB statistical decomposition indicates that approximately 20.6% of the total unadjusted association between secondary education and infant survival is shared with the proximate factors. However, the remaining portion of the association (79.4%) is left unexplained by our covariates and mediators. This remaining share may represent soft maternal skills such as baseline nutritional knowledge, localized family bargaining power, or unobserved material resources, but it is also highly likely to be a reflection of severe unmeasured confounding.

Because our study relies on retrospective cross-sectional survey data (SLHDS 2020), the classical assumptions required for causal mediation analysis (sequential ignorability) are highly unlikely to be satisfied. We must systematically discuss how these assumptions are challenged in this setting by important unmeasured confounders: (1) Exposure-Outcome Confounding (Maternal Education → Infant Survival): Important pre-exposure variables that simultaneously shape a woman’s educational attainment and her infant’s subsequent survival are omitted from our models due to data constraints. These include maternal childhood socioeconomic status, parental education, maternal childhood health, and baseline cognitive ability. For example, a woman raised in a wealthy, well-resourced urban household is highly likely both to complete secondary school and to experience superior child survival outcomes, regardless of her own education. (2) Mediator-Outcome Confounding (Mediators → Infant Survival): The relationship between our proximate mediators (especially skilled birth attendance and environmental sanitation) and infant survival is heavily exposed to unmeasured confounding. A critical missing variable is the geographic distance or travel time to health facilities. Families living closer to urban centers or functional health clinics have both higher rates of utilizing skilled delivery and lower infant mortality due to overall physical access to emergency care. Similarly, the clinical quality of local health clinics—such as the availability of emergency drugs, electricity, and clean surgical equipment—is unmeasured. This directly confounds the relationship between skilled delivery and infant survival. (3) Exposure-Mediator Confounding (Maternal Education → Mediators): The decision to seek skilled birth attendance is influenced by unmeasured baseline factors such as maternal pre-exposure health literacy, traditional family structures, and paternal support. Husbands who are highly supportive or more educated may facilitate both the mother’s continuing education and her utilization of professional obstetric care, confounding the direct link between maternal education and skilled delivery. (4) Mediator-Outcome Confounders Affected by Exposure (Maternal Education → Confounder → Mediators → Infant Survival): Post-education household decisions—such as subsequent marriage dynamics, maternal labor force participation, and maternal income generation—might act as intermediate confounders. These factors are shaped by maternal education and simultaneously influence both her access to skilled delivery and her infant’s survival.

Furthermore, we must account for omitted biological and socio-demographic parameters: (1) Biodemographic Risks (Maternal Age, Birth Order, Birth Interval, Child Sex, and Multiple Births): Biological and demographic characteristics are highly influential in predicting early-life survival. For instance, multiple births (twins/triplets) and children born to very young mothers (< 18 years) face substantially higher physiological risks of neonatal and infant mortality. Firstborns and children of extremely high birth orders are also statistically exposed to elevated hazards. By omitting these variables, our Cox models may experience misspecification, potentially overestimating or underestimating the baseline hazard of infant death. (2) Socioeconomic and Household Risks (Maternal Employment, Paternal Education, and Marital Status): Paternal education is a recognized driver of household resource allocation and health-seeking behaviors. Similarly, maternal employment determines the household’s disposable income and maternal time-allocation for infant care. Marital status serves as a proxy for social, emotional, and economic support during pregnancy and postpartum. The exclusion of these social factors limits our capacity to account for paternal influence and maternal time constraints, leaving some portion of the unadjusted educational threshold’s effect uncaptured.

While we recognize the statistical consequences of these omissions, including these variables in the primary SLHDS analysis was methodologically unfeasible due to severe missingness (paternal education/maternal employment), structural selection bias (birth intervals), and highly skewed distributions (marital status). Consequently, our estimates represent descriptive, unadjusted household-level associations. We strongly caution that future work with more complete longitudinal datasets is required to control for these variables and establish true structural pathways.

A notable challenge in our analysis is that only six infant deaths occurred among mothers with secondary education or higher. While this small event count reflects a descriptive protective advantage, it exposes our standard maximum likelihood Cox regression to sparse-data bias (or finite sample bias), which can generate inflated or unstable hazard ratios and artificially distort mediation decompositions (37). Standard Cox models assume adequate event numbers in each covariate class; when this assumption is violated, standard errors can be underestimated and coefficients can be biased away from the null. We addressed this directly by running Firth’s Penalized Likelihood Cox Regression as a sensitivity analysis. The penalized likelihood estimates yielded hazard ratios (Unadjusted HR = 0.358; Adjusted HR = 0.521) that were virtually identical to our maximum likelihood estimates. This consistency suggests that our primary results do not suffer from severe separation or monotone likelihood issues. Furthermore, collapsing the exposure into a binary classification yielded equivalent patterns of baseline significance and complete adjustment-induced attenuation, reinforcing the robustness of our descriptive threshold conclusion.

Our strict definition of “better” ANC (initiated in the first trimester with at least four visits) restricted this category to only 1.2% of the analytical sample. While theoretically aligned with WHO guidelines, this restrictive cut-off resulted in low statistical power, as reflected in the extremely wide confidence intervals for ANC in our fully adjusted models (Model 7 HR = 0.95, 95% CI: 0.17–5.18). Our sensitivity analyses using less restrictive, standard ANC definitions (‘Any ANC vs. None’ and ‘Four or more visits regardless of timing’) successfully resolved this power constraint, yielding significantly tighter confidence intervals and demonstrating a more stable baseline relationship with child survival. Furthermore, these alternative definitions revealed a slightly larger descriptive mediation share for antenatal care (6.4 and 7.8% respectively, compared to 3.2% in our primary analysis). This indicates that while antenatal care indeed plays an active role in accounting for the educational threshold’s association, it remains secondary to skilled birth attendance, which consistently emerged as the single most critical proximate pathway across all specifications.

A critical methodological insight provided by our causal DAG (Figure 2) is that current household wealth, place of residence, and subnational region ( Z ) represent post-exposure intermediate characteristics that are partially determined by maternal education. In traditional modeling, these variables are adjusted for as baseline confounders. However, in our structural framework, Z represents an intermediate block on the pathway between education ( X ) and infant survival ( Y ). By controlling for Z in Model 2, we block the causal flow X→Z→Y and X→Z→M→Y , inducing overadjustment bias. This blocking explains why the unadjusted protective association of secondary education (Model 1 HR = 0.343, p<0.05 ) is completely attenuated and loses significance immediately upon introducing these intermediate covariates in Model 2 (HR = 0.419, p=0.063 ). This attenuation does not mean that maternal education is irrelevant to child survival; rather, it suggests that the majority of education’s descriptive association operates precisely by elevating household socioeconomic status and enabling migration to urban areas or regions with superior health infrastructure. Consequently, Model 7 must not be interpreted as the ‘true’ causal effect of education, but rather as a highly conservative estimation of the residual direct association of education after holding current household resources and geographic location constant.

Interestingly, environmental factors like clean water and sanitation provided negligible mediation (1.3%). This finding diverges from the classic “proximate determinants” framework by Mosley and Chen (24), which emphasizes environmental contamination as a primary pathway. In Somaliland, the intervention of a skilled attendant appears to share a much stronger association with infant survival than the household’s physical environment, likely due to the high prevalence of birth-related complications in the region (22).

The study highlighted significant geographic and economic disparities. The Togdheer region exhibited a mortality rate of 85 per 1,000, nearly double that of Awdal (42 per 1,000). These disparities persist even when controlling for education, suggesting that regional infrastructure and health deserts play a role that individual education cannot fully offset (27, 28). This massive regional disparity can be explained by differences in geography, infrastructure, and services. Togdheer is characterized by vast, arid, nomadic terrains with highly dispersed populations, severe transportation barriers, and a lack of functional obstetric services. In contrast, Awdal features a more urbanized population with relatively better-integrated community health networks and midwifery training programs linked to local institutions (10).

Furthermore, while descriptive statistics showed a slightly higher infant mortality rate in the middle wealth tertile compared to the poorest, this difference did not persist in our adjusted multivariate models. The hazard ratio for the middle-wealth category was close to unity and statistically nonsignificant across all adjusted models. This indicates that the descriptive disparity is likely due to confounding factors—such as regional composition or urban–rural distribution—or potential measurement limitations within the wealth index itself, rather than a systematic wealth-based disadvantage.

These results have implications for the 2030 Agenda. While SDG 3.2 targets a reduction in neonatal mortality to at least 12 per 1,000 live births and under-5 mortality to at least 25 per 1,000 live births, our recorded infant mortality rates (ranging sub-nationally from 42 to 85 per 1,000 live births) indicate that the region faces a substantial challenge in meeting these child survival targets. Our finding that education is associated with infant survival primarily through its link with skilled delivery suggests that progress toward SDG 3 may benefit from recognizing the synergies between SDG 4 (Quality Education) and SDG 3 (Good Health) (18). Specifically, female transition to secondary school should be viewed as a public health priority (21, 29).

The statistical mediation analysis using the KHB method rests on the strong assumption of no unmeasured confounding of the exposure-outcome, exposure-mediator, and mediator-outcome relationships (30). Given the cross-sectional and observational nature of the SLHDS data, these assumptions are unlikely to hold completely. Unobserved maternal characteristics—such as innate health literacy, baseline maternal physical health, physical distance to health facilities, or localized cultural norms regarding healthcare utilization—could confound these relationships. For instance, families living closer to well-resourced facilities may be more likely to have highly educated mothers and better access to skilled delivery, creating a spurious correlation. Consequently, our mediation estimates should be interpreted as descriptive decompositions rather than definitive causal pathways. While the cross-sectional nature of the SLHDS limits direct causal claims, the use of Cox proportional hazards and KHB mediation provides an informative estimation of these statistical associations for this region.

Finally, our retrospective design is susceptible to survival and recall bias. First, because the SLHDS only interviews surviving mothers, infants of deceased mothers—who face a substantially higher risk of mortality—are systematically excluded from the sample, potentially underestimating the true mortality hazard. Second, systematic recall bias may exist if maternal education relates to the accuracy of reporting early neonatal deaths, with less-educated mothers potentially underreporting stillbirths or early deaths. These limitations are inherent to retrospective cross-sectional demographic surveys in low-resource and fragile settings.

### 4.1 Strengths of the study

1. Nationally representative dataset: This study utilizes the 2020 Somaliland Health and Demographic Survey (SLHDS), providing a large, nationally representative sample that accounts for the complex survey design (clustering, stratification, and sampling weights) to produce generalizable sub-national estimates.
2. Methodological rigor: By utilizing Cox Proportional Hazards models combined with the Karlson–Holm–Breen (KHB) mediation method, this study represents the first formal decomposition of maternal education’s direct and indirect associations with infant survival in Somaliland.
3. Formal threshold testing: Unlike previous studies that only compare education levels against a baseline of “no education,” this study implements post-estimation Wald tests to formally evaluate the contrast between primary and secondary education, providing empirical evidence for the threshold hypothesis.

### 4.2 Study limitations

1. Cross-sectional and observational design: Because the SLHDS is a cross-sectional survey, temporal order cannot be definitively established, which limits our ability to make direct causal claims.
2. Endogeneity and omitted variable bias: Maternal education is treated as exogenous, but it may be jointly determined by unobserved childhood socioeconomic status, regional infrastructure, or family background. Since we control only for post-exposure characteristics, the estimated associations may be subject to residual confounding.
3. Unmeasured confounding in mediation: The KHB mediation analysis assumes no unmeasured confounding among the education, mediator, and survival relationships. Unobserved maternal traits, such as physical distance to health clinics or innate health literacy, could confound these pathways.
4. Statistical power constraints for ANC: The strict clinical definition of “better” antenatal care (initiated in the first trimester with at least four visits) restricted this category to 1.2% of the sample, resulting in wide confidence intervals and a potential lack of statistical power to detect mediation effects for this specific pathway.
5. Recall and survival bias: Retrospective reporting of births over 5 years is subject to maternal recall bias. Additionally, because only surviving mothers were interviewed, the sample excludes infants of deceased mothers, who are statistically at higher risk of mortality.

### 4.3 Policy implications

1. Prioritize female transition to secondary education: Since primary education alone did not demonstrate a descriptive or analytical survival advantage, educational policies in Somaliland must go beyond basic primary enrollment. Efforts should focus on systemic interventions such as targeted secondary school bursaries for girls that reduce attrition and facilitate female transition to, and completion of, secondary school.
2. Strengthen and expand skilled obstetric services: Because skilled birth attendance is the primary pathway mediating the education-survival relationship, direct investments must be made to train, deploy, and retain midwives and skilled birth attendants, particularly in rural areas and geographic “health deserts” such as Togdheer by expanding the network of Basic Emergency Obstetric and Newborn Care (BEmONC) facilities.
3. Bridge the education-utilization gap: Since the protective effect of secondary education operates indirectly through service utilization, health campaigns utilizing radio and mobile SMS should target less-educated mothers with accessible health literacy tools, lowering the behavioral barriers to seeking professional delivery care.
4. Develop targeted regional interventions: Given the stark regional disparities (mortality ranging from 42 per 1,000 in Awdal to 85 per 1,000 in Togdheer), resource allocation for maternal and child health infrastructure should be geographically prioritized to target high-burden regions such as rural Togdheer, focusing on mobile health clinics and emergency referral transport.

## 5 Conclusion

In conclusion, secondary education was associated with lower infant mortality in unadjusted analysis, but this association was attenuated and became statistically non-significant after adjustment. The observed educational gradient appears largely explained by skilled birth attendance in descriptive decomposition analyses. While maternal education remains a vital developmental tool, achieving Sustainable Development Goal 3.2 in Somaliland will require integrated, realistic policies that simultaneously promote female secondary education and expand physical and economic access to professional obstetric care in historically underserved subnational regions.

## Data Availability

The datasets analyzed during the current study are available in the Demographic and Health Surveys (DHS) Program repository, https://dhsprogram.com/data/available-datasets.cfm. Specifically, the 2020 Somaliland Health and Demographic Survey (SLHDS) data were used.
