# Supplementary material for: Maternal education and infant survival in Somaliland: a descriptive decomposition of observed survival differences across sequential adjustment models
Source: Front Public Health. 2026 Jul 29;14:1886814. doi: 10.3389/fpubh.2026.1886814 (PMC13461726; doi:10.3389/fpubh.2026.1886814)
Supplement: Supplementary file 1 [file Table_1.docx]

**Supplementary Table S1. Sensitivity analyses assessing the robustness of the association between maternal education and infant mortality**

| **Sensitivity model** | **Comparison** | **Adjusted HR** | **95% CI** | **P-value** |
| --- | --- | --- | --- | --- |
| **Model A. Standard Cox proportional hazards model (Primary analysis)** | Secondary education vs. no formal education | 0.501 | 0.20–1.26 | 0.140 |
|  | Primary education vs. no formal education | 1.096 | 0.66–1.82 | 0.724 |
| **Model B. Firth penalized Cox regression** | Secondary education vs. no formal education | 0.521 | 0.22–1.21 | 0.131 |
|  | Primary education vs. no formal education | 1.074 | 0.65–1.76 | 0.778 |
| **Model C. Binary maternal education coding** | Secondary/higher vs. primary or no formal education | 0.518 | 0.22–1.21 | 0.129 |
| **Model D. Alternative ANC definition** | Any ANC visit vs. no ANC visit | 0.824 | 0.55–1.23 | 0.344 |
| **Model E. Alternative ANC definition** | ≥4 ANC visits regardless of timing vs. <4 visits | 0.768 | 0.46–1.28 | 0.312 |

*(Tip: Make sure to remove the table from the main manuscript file if the journal requires it to only appear in the separate supplementary file.)*
